# Theoretical Methods for Assessing the Density of Protein Nanodroplets

**DOI:** 10.3390/ijms26178631

**Published:** 2025-09-04

**Authors:** Midhun Mohan Anila, Michał Wojciechowski, Mateusz Chwastyk, Bartosz Różycki

**Affiliations:** Institute of Physics, Polish Academy of Sciences, Al. Lotników 32/46, 02-668 Warsaw, Poland; midhun@ifpan.edu.pl (M.M.A.); mwoj@ifpan.edu.pl (M.W.); chwastyk@ifpan.edu.pl (M.C.)

**Keywords:** LLPS, protein nanodroplets, α-synuclein, MD simulations, coarse-grained models, SPACEBALL

## Abstract

Many intrinsically disordered proteins (IDPs) are known to undergo liquid–liquid phase separation (LLPS), which is a physical process that drives the formation of biomolecular condensates and membraneless organelles in biological cells. Molecular dynamics (MD) simulations provide valuable tools to explore both the molecular mechanisms of LLPS and the physical properties of biomolecular condensates. However, a direct comparison of MD simulation results with phase diagrams obtained experimentally is normally prevented not only by the high computational costs of simulating large biomacromolecular systems on sufficient timescales but also by conceptual challenges. Specifically, there currently seems to be no standard or unambiguous method of defining and determining volumes occupied by coexisting phases at the nanoscale, with typically no more than a few hundred biomacromolecules in the simulation box. The goal of this work is to fill in this gap in the methodology. Focusing on α-synuclein as a model IDP, we test and compare three methods for determining the molecular density of protein nanodroplets, or clusters, generated in MD simulations or using other molecular modeling approaches. Two of the methods are based on approximating nanodroplets with homogeneous spheres and ellipsoids, respectively. The third method, which is expected to yield the most physically accurate results, is based on the SPACEBALL algorithm, with optimized, cluster-specific radii for volume probes. Our results contribute to the construction of accurate phase diagrams on the basis of MD simulations of IDP systems.

## 1. Introduction

Liquid–liquid phase separation (LLPS) is a fundamental biophysical process in which proteins and other biomolecules demix from a homogeneous solution to form liquid droplets [1]. This phenomenon plays a crucial role in cellular organization, enabling the formation of membraneless organelles such as stress granules, nucleoli, and P-bodies, which regulate various biological processes, including RNA metabolism, signal transduction, and protein homeostasis [2,3,4]. LLPS is driven primarily by multivalent interactions between intrinsically disordered regions and modular protein domains, and can be influenced by such physical factors as temperature, pH, salt concentration, and molecular crowding [5].

A crucial tool for understanding the conditions under which phase separation occurs, as well as the physical properties of the resulting phases, is the phase diagram [6]. A typical phase diagram for LLPS maps parameters such as temperature, protein concentration, and salt concentration, delineating regions where a homogeneous mixture exists and where phase separation leads to the formation of biomolecular condensates [7,8,9]. The binodal curve defines the boundary between the single-phase and two-phase regions, while the spinodal region represents conditions where phase separation occurs spontaneously via nucleation-independent mechanisms [10,11]. Understanding such phase diagrams is essential for deciphering cellular compartmentalization mechanisms and developing strategies to modulate phase behavior in disease contexts.

Several methods are used to construct phase diagrams, including experimental, theoretical, and computational approaches. Experimental techniques involve differential scanning calorimetry [12,13], X-ray diffraction [14], and optical or electron microscopy [15] to identify phase transitions and equilibrium states. Thermodynamic modeling, such as the CALPHAD (Calculation of Phase Diagrams) method [16,17,18], combines experimental data with thermodynamic equations to predict phase diagrams. Computational methods, including molecular dynamics (MD) [19] and density functional theory [20], can provide insights into phase stability at the molecular scale, even for complex biomacromolecular systems. However, constructing phase diagrams for biomacromolecular systems on the basis of MD simulations is a very difficult task, not only because of the high computational costs but also due to conceptual challenges related to finite-size effects. To the best of our knowledge, there is currently no standard or unambiguous method of defining and determining the volumes occupied by coexisting phases at the nanoscale with typically no more than a few hundred biomacromolecules in the simulation box. Our objective is to fill this gap in the methodology by presenting a well-defined and robust method of computing the volumes and densities of protein nanodroplets or clusters generated in MD simulations or by other molecular modeling techniques.

Here, we test and assess different methods for calculating the molecular density of nanodroplets, or clusters, formed by intrinsically disordered proteins (IDPs). For the purpose of this study, we use α-synuclein, which is a physiologically relevant and well-studied IDP known to undergo LLPS at certain conditions [21]. Specifically, α-synuclein is a 140-residue IDP abundantly expressed in the mammalian brain, where it functions primarily at the presynaptic terminal to regulate synaptic vesicle trafficking, neurotransmitter release, and lipid interactions [22,23]. Its chaperone-like activity in modulating SNARE complex assembly further underscores its central role in maintaining synaptic functions [22]. Under physiological conditions, α-synuclein remains conformationally dynamic and lacks a stable secondary structure; however, it adopts an α-helical conformation upon membrane binding, with residues 3-37 and 45-92 forming curved α-helices, as revealed by NMR structural studies [24]. The relevance of this dynamic behavior is reinforced by MD simulations, which consistently depict α-synuclein as highly disordered, exhibiting persistent secondary-structure fluctuations and minimal propensity for β-sheet formation in its monomeric form [25,26,27,28].

The LLPS of α-synuclein is primarily mediated by weak multivalent interactions involving the NAC domain and the acidic C-terminal region, and is proposed to be critical for normal cellular functions, including synaptic organization, while also serving as a potential precursor to pathological aggregation into amyloid fibrils [29,30,31]. Depending on experimental conditions (such as salt concentration, temperature, pH value, and polyethylene glycol (PEG) concentration), the LLPS of α-synuclein has been observed in a broad range of protein concentrations, between 15 and 500 μM [31,32,33]. The concentration of α-synuclein within droplets has been found to range between 260 and 600 μM in the presence of 10% PEG [34]. However, some publications report no LLPS of α-synuclein at protein concentrations of up to 500 μM at pH 7.4 [35].

The amount of research on α-synuclein, as well as the physiological relevance of this protein, provide a rationale for selecting clusters of α-synuclein molecules—generated either in MD simulations or using other molecular modeling approaches—as model systems for developing and evaluating methods for protein cluster density determination. Among the methods evaluated in this study, the one that is expected to yield the most physically accurate results is based on the SPACEBALL algorithm, which was originally developed for geometrical analyses of protein structures with cavities [36]. Importantly, the original parametrization of SPACEBALL is suitable for computing cavity volumes in protein structures and not for determining densities of IDP clusters. Here, we present a robust approach to optimizing the SPACEBALL parameters for any given IDP cluster, which enables cluster density computation with satisfactory precision, which is a critical step toward constructing accurate phase diagrams.

Recently, a SPACEBALL-based approach has been introduced to construct a phase diagram of the van der Waals fluid [37]. This approach involves a combination of three key elements: (i) counting free molecules that are not bound to any cluster, (ii) computing the density of the largest cluster, and (iii) calculating the density of the entire system. The high-density branch of the binodal line is then determined from the number of free molecules not in contact with any cluster. In contrast, the low-density branch of the binodal line is obtained from the SPACEBALL-calculated densities. Specifically, the position of the low-density branch is identified by analyzing the system density at which fluctuations in the largest cluster density become apparent. Moreover, the spinodal line is determined by monitoring the system-wide density variations. The onset of strong fluctuations indicates the left spinodal boundary, while their disappearance marks the right spinodal branch. The main purpose of our present work is to adapt and re-parameterize the SPACEBALL-based method of density calculation to systems of IDPs, with the aim of applying this method in future studies to explore phase diagrams of IDPs.

Our SPACEBALL-based method of computing protein nanodroplet density is clearly distinct from the slab method, which is commonly used in the MD community to determine the densities of two coexisting phases in polymer or IDP systems [38,39]. In the framework of the slab method, MD simulations are performed using a cuboid box of side lengths $L_{x}$, $L_{y}$ and $L_{z}$, where $L_{z}$ is taken to be much larger than $L_{x}$ and $L_{y}$. The polymers or IDPs under study are prearranged into a slab perpendicular to the *z* axis and spanning through the simulation box in the *x* and *y* directions. Due to the periodic boundary conditions applied in all three directions of the simulation box, the slab is typically stable during the MD simulations. After equilibration, the slab is taken as the condensed phase, whereas the IDPs in the remaining part of the simulation box represent the dilute phase. The condensed phase density, as well as the dilute phase density, is obtained from the time-averaged density profile along the *z*-axis. These densities can then be used to construct the phase diagram.

Although the slab method is useful and relatively simple to implement, it suffers from a few drawbacks. Firstly, there is no formula or protocol for selecting the box side lengths: $L_{x}$, $L_{y}$, and $L_{z}$. However, the choice of the box side lengths is relevant as it determines both the time of equilibration and the thickness of the slab at equilibrium, and thus can influence the estimation of the densities of the two coexisting phases. Secondly, it is assumed that the slab remains perpendicular to the *z* axis throughout the simulation. However, thermal fluctuations can cause bending or shear deformations in the slab during the simulation, thus affecting the time-averaged density profile along the *z* axis, which can lead to errors in the estimation of the densities of the two phases. Thirdly, when density fluctuations are large (e.g., in the vicinity of the critical point), the slab interface is rough and cavities can form spontaneously inside the slab, giving rise to significant uncertainties of the time-averaged density profile along the *z* axis. On the other hand, none of these issues pertain to our SPACEBALL-based method, simply because our method does not impose any restrictions on the shape of IDP nanodroplets condensates. Thus, our SPACEBALL-based method seems to be more robust, geometry-independent, and less sensitive to interface fluctuations than the slab method.

## 2. Results

We performed coarse-grained MD simulations of fifty molecules of α-synuclein at three temperatures, as described in Section 4.1. Then, we used a contact-based method to identify α-synuclein clusters in the MD trajectories, and selected two sets of geometrically diverse clusters for further analysis, as described in Section 4.2. Additionally, as described in Section 4.3, we introduced three groups of clusters with well-defined volumes and shapes (namely, spheres, ellipsoids, and cylinders) for assessments of density determination methods. The spherical, ellipsoidal, and cylindrical clusters were constructed using Packmol software [40] and provided reference density values against which the calculation results could be validated.

In this section, we discuss three methods for density calculation. Each of the methods is introduced, applied, and discussed in a separate subsection below. The first two of the methods are based on approximating molecular clusters by homogeneous spheres and ellipsoids, respectively, as detailed in Section 2.1 and Section 2.2. These methods are rather conceptually simple and computationally fast. The third method is computationally more demanding and time-consuming as it employs the SPACEBALL software [28,36]. The method boils down to identifying optimal parameters for SPACEBALL computations of protein cluster volumes, as discussed in Section 2.3.

### 2.1. Spherical Approximation

**Figure 1:**
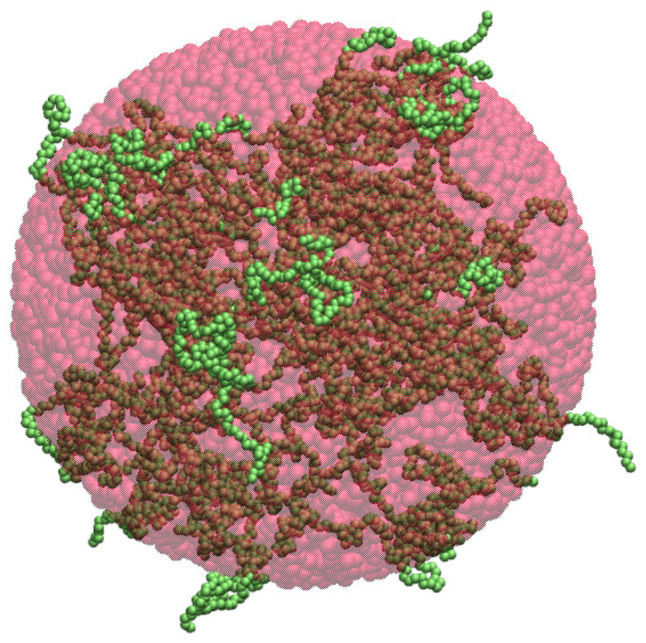
Illustration of the spherical approximation for the cluster density calculation. The α-synuclein chains forming a cluster are shown in green and a homogeneous sphere approximating the cluster’s volume is semi-transparent and colored in red. Importantly, the sphere has the same radius of gyration as the cluster.

The radius of gyration, denoted herein by $R_{g}$, is often used in polymer physics to describe the dimensions of polymer chains. It is straightforward to compute the radius of gyration for each of the protein clusters introduced in Section 4.2 and Section 4.3. The resulting $R_{g}$ values can then be used to estimate the cluster density. Firstly, the shape of a given cluster is approximated by a sphere whose mass distribution is uniform and has a gyration radius equal to the $R_{g}$ value of the cluster, as illustrated in Figure 1. The radius of such a sphere is $R=R_{g}\sqrt{5/3}\approx 1.291\;R_{g}$ and its volume is $V_{S}=4\pi R^{3}/3\approx 9.013\;R_{g}^{3}$. Then, the density of the cluster is taken as $d=N/V_{S}\approx 0.111\;N/R_{g}^{3}$, where *N* denotes the total number of residues forming the cluster and $R_{g}$ is the radius of gyration of the cluster.

**Figure 2:**
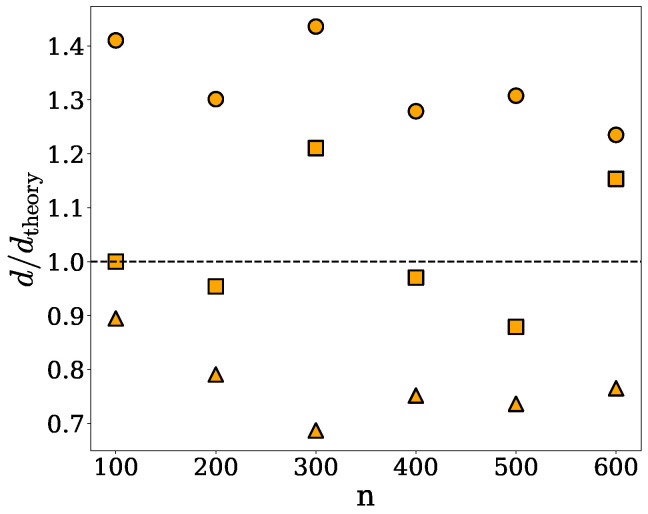
The density ratio $d/d_{\mathrm{theory}}$ *versus* the number *n* of chains in a given cluster of the spherical (circles), ellipsoidal (squares), or cylindrical (triangles) shape. Here, $d_{\mathrm{theory}}$ and *d* denote, respectively, the theoretical density specified in Section 4.3 and the density computed under the spherical approximation.

For the purpose of this study, we compare the densities obtained for the spherical, cylindrical, and ellipsoidal clusters under the spherical approximation, *d*, with their respective theoretical values, $d_{\mathrm{theory}}$, introduced in Section 4.3. Specifically, Figure 2 shows the density ratio $d/d_{\mathrm{theory}}$ *versus* the number *n* of chains in a given cluster of the spherical, ellipsoidal, or cylindrical shape. Since the spherical approximation is based on the assumption of uniform mass distribution, it overestimates the densities of spherical clusters in which protein chains are distributed randomly but not entirely homogeneously. On the other hand, the spherical approximation does not capture the elongated shape of the cylindrical clusters, underestimating their densities. These two effects cancel out to some degree for the ellipsoidal clusters, for which *d* is in agreement with $d_{\mathrm{theory}}$ within less than about 20%.

The discrepancies between the theoretical and computed densities, as seen in Figure 2, arise because the radius of gyration represents the mass-weighted average distance from the center of mass, rather than the true spatial extent of the cluster. As a result, the approximated spherical volume does not capture the actual volume occupied by the cluster, particularly in the case of highly irregular or loosely connected structures. Consequently, the density calculations based on this approach do not accurately reflect the physical extension of the clusters, as illustrated in Figure 1, where an α-synuclein cluster (shown in green) is approximated by a sphere (shown in red) whose radius of gyration equals the cluster’s radius of gyration. The spherical approximation fails to encompass several regions of the cluster that extend beyond the spherical boundary. This example underscores the inability of this method to capture shape anisotropy and non-uniform mass distribution, rendering it unreliable for estimating the density of irregular-shaped clusters. In addition, cavities or voids between chains or sub-clusters are not taken into account under the spherical approximation, which can result in an underestimation of the cluster volume and, consequently, an overestimation of the density. Nevertheless, the densities obtained under the spherical approximation can provide information about the compactness and packing of protein chains within the clusters.

### 2.2. Ellipsoidal Approximation

The spherical approximation, based on the concept of the radius of gyration, is not well-suited for protein clusters with non-globular or irregular shapes, as indicated in the previous subsection. To address this limitation, we now employ an alternative method [41] based on the tensor of inertia, which offers a compact representation of the mass distribution relative to rotational axes. Although the tensor of inertia can be computed with respect to any point in space, it is most commonly calculated around the center of mass. Then, for a system of *N* point masses, the tensor of inertia is defined as

(1) $$I=\left[\begin{array}{ccc} I_{xx} & I_{xy} & I_{xz}\\ I_{yx} & I_{yy} & I_{yz}\\ I_{zx} & I_{zy} & I_{zz} \end{array}\right]$$

with diagonal elements, representing the moments of inertia about the coordinate axes, given by

(2) $$I_{xx}=\sum_{k=1}^{N}\left(y_{k}^{2}+z_{k}^{2}\right),\;I_{yy}=\sum_{k=1}^{N}\left(x_{k}^{2}+z_{k}^{2}\right),\;I_{zz}=\sum_{k=1}^{N}\left(x_{k}^{2}+y_{k}^{2}\right)$$

and off-diagonal elements, also called the products of inertia, computed as

(3) $$I_{xy}=I_{yx}=-\sum_{k=1}^{N}x_{k}y_{k},\;I_{xz}=I_{zx}=-\sum_{k=1}^{N}x_{k}z_{k},\;I_{yz}=I_{zy}=-\sum_{k=1}^{N}y_{k}z_{k}$$

**Figure 3:**
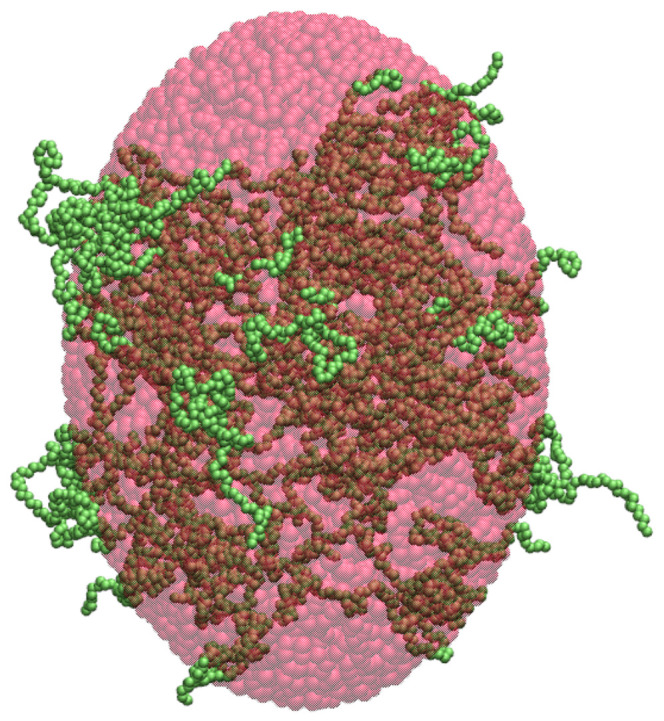
Illustration of the ellipsoidal approximation for the cluster density calculation. The α-synuclein chains forming a medium-size cluster are shown in green. The homogeneous ellipsoid with semi-axis lengths derived from the cluster’s principal moments of inertia is semi-transparent and shown in red.

The tensor of inertia can be computed using Equations (1)–(3) and then diagonalized for each of the protein clusters introduced in Section 4.2 and Section 4.3. The resulting eigenvalues $\lambda_{x}$, $\lambda_{y}$ and $\lambda_{z}$ determine the principal moments of inertia, corresponding to the axes along which the cluster has no products of inertia. An ellipsoid with a uniform mass distribution and the principal moments of inertia equal $\lambda_{x}$, $\lambda_{y}$, and $\lambda_{z}$ has the semi-axes lengths $R_{x}=\sqrt{5\left(\lambda_{y}+\lambda_{z}-\lambda_{x}\right)/\left(2N\right)}$, $R_{y}=\sqrt{5\left(\lambda_{x}+\lambda_{z}-\lambda_{y}\right)/\left(2N\right)}$ and $R_{z}=\sqrt{5\left(\lambda_{x}+\lambda_{y}-\lambda_{z}\right)/\left(2N\right)}$ [42]. Here, the cluster shape is approximated by such an ellipsoid with volume $V=4\pi R_{x}R_{y}R_{z}/3$, as illustrated in Figure 3. Therefore, the cluster density is estimated as $d=3N/(4\pi R_{x}R_{y}R_{z})$, where *N* is the total number of residues in the cluster.

The accuracy of the density estimation depends sensitively on how well the ellipsoidal model captures the actual shape of a given cluster. Furthermore, under the ellipsoidal approximation, internal cavities or voids between chains or sub-clusters are not taken into account, which can result in an underestimation of the cluster volume and, consequently, an overestimation of the density. Nevertheless, the densities obtained within the ellipsoidal model provide a measure of how compactly the residues are packed within the clusters.

**Figure 4:**
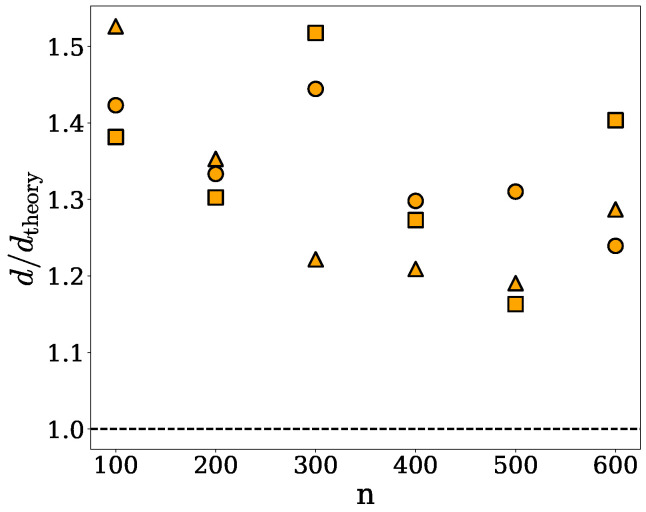
The density ratio $d/d_{\mathrm{theory}}$ *versus* the number *n* of chains, forming a given cluster of the spherical (circles), ellipsoidal (squares) or cylindrical (triangles) shape. Here, $d_{\mathrm{theory}}$ and *d* denote, respectively, the theoretical density specified in Section 4.3 and the density computed under the ellipsoidal approximation.

The data shown in Figure 4 provide a comparison of the theoretical densities of the spherical, ellipsoidal and cylindrical clusters, $d_{\mathrm{theory}}$, with the corresponding densities obtained under the ellipsoidal approximation, *d*. Specifically, Figure 4 shows the density ratio $d/d_{\mathrm{theory}}$ *versus* the number *n* of chains per cluster. As one would expect, the ellipsoidal and spherical approaches yield identical results for each of the spherical clusters. Moreover, the ellipsoidal approximation is seen to systematically overestimate the density of clusters of all shapes.

Although the ellipsoidal method shares many of the limitations of the spherical approach, it is expected to yield more accurate density estimates for elongated or anisotropic clusters, as their shapes are generally better approximated by ellipsoids. Major limitations of this method are illustrated in Figure 3, which shows an α-synuclein cluster (green) enclosed within an ellipsoid (red) whose semi-axes correspond to the principal dimensions derived from the moment of inertia of the cluster. As can be seen in Figure 3, the ellipsoid fails to fully capture the actual volume of the cluster, particularly in regions with a non-uniform mass distribution or internal cavities. This geometric mismatch contributes to the observed overestimation of the computed densities, as seen in Figure 4.

### 2.3. SPACEBALL

The density of protein clusters can also be computed using the SPACEBALL software [36] (version 2.0). Although the SPACEBALL calculation is computationally more demanding than the approximations presented in Section 2.1 and Section 2.2, it is expected to yield the most accurate results for protein clusters with irregular shapes.

The SPACEBALL algorithm combines grid-scanning and sphere-filling techniques [36]. The volume calculation starts with enclosing a given protein cluster by a cuboid box. Next, a cubic grid with the lattice constant *a* is generated within this box. The default value of the lattice constant, a=0.3 nm, roughly corresponds to the water molecule diameter. Next, spherical probes are placed at the lattice points that delineate the walls of the box. Then, the walls are moved inward along their normal directions—corresponding to the Cartesian axes—in discrete steps equal to the lattice constant *a*. As the probes move inward, each sphere is checked for overlap with protein atoms. If a probe overlaps with any atom of the protein cluster, its movement is stopped, while other probes on the same wall continue moving until they also encounter an overlap with protein atoms. The positions of non-overlapping probes are recorded on the lattice. When a probe overlaps with at least one atom of the protein cluster, its position is marked differently. This marking procedure leads to a simple classification of the lattice points: those with no contact are considered outside the protein, while those that encounter the protein cluster define its surface. In addition, the unmarked lattice points within the protein-cluster boundary define its interior volume.

A key parameter of the SPACEBALL method is the radius $r_{\mathrm{probe}}$ of the wall probes. This parameter governs the resolution of surface detection and, consequently, the accuracy of the volume estimate. Importantly, the optimal radius of the wall probes depends on the shape and density of the cluster under study. Namely, if the probe radius is too small, the probes may penetrate the cluster interior, creating artificial cavities and underestimating the cluster volume. Conversely, if the probe radius is too large, it may overestimate the cluster volume by including artificial ‘pockets’ on the cluster surface, thereby artificially enlarging the cluster. Therefore, optimizing the probe radius is critical for the accuracy of the volume calculation method. Here, we introduce a general and robust approach to identify an optimal probe radius based on the geometric properties of the cluster.

**Figure 5:**
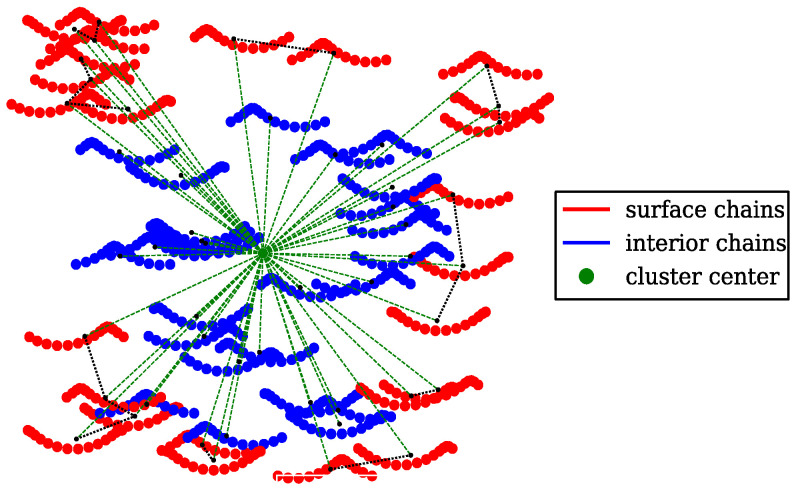
Schematic illustration of how the optimal probe radius is chosen for the cluster volume calculation using SPACEBALL. Each of the proteins forming the cluster is depicted as a chain of beads. The surface chains are marked in red. The remaining chains, marked in blue, form the interior of the cluster. The distinction between the surface chains and the interior chains is made on the basis of the distances from each of the chains to the geometrical center of the cluster. These distances are indicated by the green dotted lines. In contrast, the distances between the geometrical centers of the surface chains and their nearest neighbors are marked as the black dotted lines. The average distance between the geometrical centers of the surface chains and their nearest neighbors is selected as the surface radius, which is denoted as $r_{\mathrm{probe}}^{S}$ and depends on the surface chain fraction ϕ. The surface radius $r_{\mathrm{probe}}^{S}$ determined for ϕ=0.5 is then taken as the optimal radius $r_{\mathrm{probe}}^{O}$ of the wall probes for the volume calculation using SPACEBALL.

Our approach for selecting the optimal probe radius is based on identifying protein chains that are located on the cluster surface, as illustrated in Figure 5. Firstly, for each of the chains in a given cluster, the mean distance of its atoms from the geometrical center of the cluster is calculated. The chains are then sorted based on these mean distances, and a set of most peripheral chains is selected as surface chains (shown in Figure 5 in red). The remaining chains (shown in Figure 5 in blue) are assumed to form the interior of the cluster. The surface chains constitute a certain fraction ϕ of all chains in the cluster. Next, the geometrical center $G_{j}$ of each of the surface chains is determined. Then, each of the surface chains is assigned its nearest neighbor chain based on minimal distances between the chain centers $G_{j}$. Finally, the average distance between the geometrical centers of the surface chains and their nearest neighbors is taken as a pre-optimal radius of the wall probes, which we denote herein as a surface radius $r_{\mathrm{probe}}^{S}$. A Python script computing $r_{\mathrm{probe}}^{S}$ for a given protein cluster is deposited on the GitHub platform; see https://github.com/midhunanila/SPACEBALL_DENSITY (accessed on 2 September 2025).

**Figure 6:**
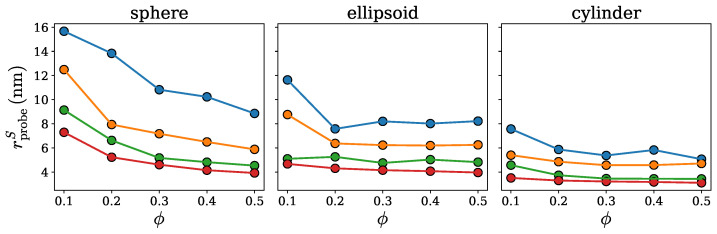
The surface radius $r_{\mathrm{probe}}^{S}$ *versus* the surface chain fraction ϕ for the spherical, ellipsoidal and cylindrical clusters with 100 (data points in blue), 200 (data points in orange), 400 (data points in green), and 600 (data points in red) chains.

The surface radius $r_{\mathrm{probe}}^{S}$ is specific for a given cluster and depends on the fraction ϕ of chains identified as the surface chains. Figure 6 shows how $r_{\mathrm{probe}}^{S}$ varies with ϕ for the spherical, ellipsoidal, and cylindrical clusters with 100, 200, 400, and 600 chains. Importantly, the surface radius is seen to be systematically larger for the loose clusters with 100 chains (data points in blue) than for the compact clusters with 600 chains (data points in red), independent of the surface chain fraction ϕ or the cluster shape. As a matter of fact, $r_{\mathrm{probe}}^{S}$ is found to monotonically decrease with an increase in the number of chains, meaning that the sparser the cluster is, the larger the selected probe radius in order to avoid penetration of the wall probes into the cluster interior. This observation is consistent with the method for identifying the surface radius of the wall probes, as described in the paragraph above.

Another relevant piece of information that follows from the results presented in Figure 6 is that the surface radius does not change significantly as the surface chain fraction ϕ varies between 0.3 and 0.5. This observation implies that the SPACEBALL calculation results should be practically unaffected by the exact value of ϕ, as long as it is in the range between 0.3 and 0.5. Therefore, in the following, we take the optimal radius $r_{\mathrm{probe}}^{O}$ of the wall probes as the surface radius $r_{\mathrm{probe}}^{S}$ at ϕ=0.5, i.e., $r_{\mathrm{probe}}^{O}=r_{\mathrm{probe}}^{S}\left(\phi =0.5\right)$. Python scripts computing the optimal probe radius for a given protein cluster are deposited on the GitHub platform; see https://github.com/midhunanila/SPACEBALL_DENSITY (accessed on 2 September 2025).

Table 1 contains the optimal values of the wall probe radius, $r_{\mathrm{probe}}^{O}=r_{\mathrm{probe}}^{S}\left(\phi =0.5\right)$, for the spherical, ellipsoidal, and cylindrical clusters. For each of these shapes, the values of $r_{\mathrm{probe}}^{O}$ are seen to decrease with an increase in the number *n* of chains in the cluster, which means that the denser the cluster is, the smaller the optimal probe radius required to properly delineate the cluster surface. For n=500 and n=600, respectively, $r_{\mathrm{probe}}^{O}\approx 3.9$ nm and $r_{\mathrm{probe}}^{O}\approx 3.8$ nm, independent of the cluster shape, indicating that the cluster density is the major factor determining the optimal probe radius in the case of sufficiently dense clusters.

**Figure 7:**
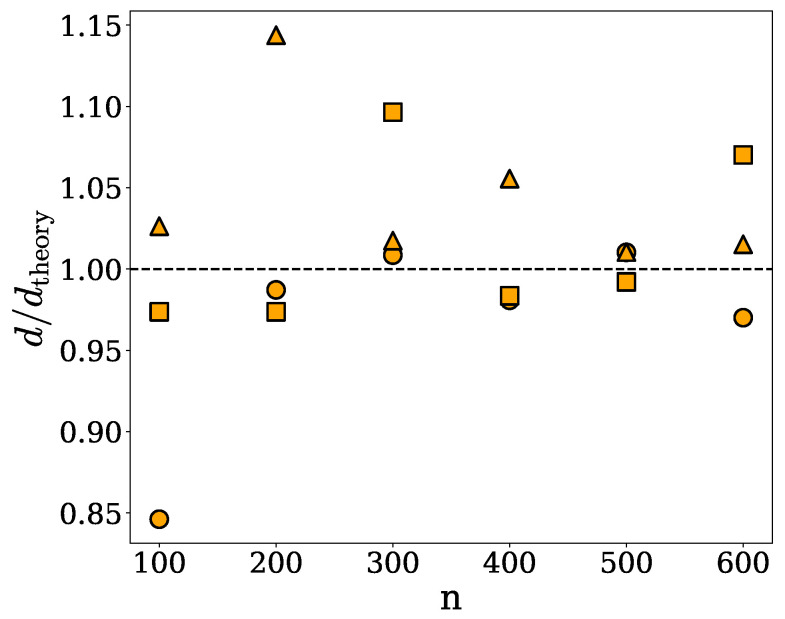
The density ratio $d/d_{\mathrm{theory}}$ *versus* the number *n* of chains in a given cluster of the spherical (circles), ellipsoidal (squares), or cylindrical (triangles) shape. Here, $d_{\mathrm{theory}}$ and *d* denote, respectively, the theoretical density specified in Section 4.3 and the density computed using SPACEBALL with the optimal probe radii given in Table 1.

Table 1 also contains the densities of the spherical, ellipsoidal, and cylindrical clusters computed using SPACEBALL with the specified values of $r_{\mathrm{probe}}^{O}$, as well as the corresponding theoretical densities $d_{\mathrm{theory}}^{S}$, $d_{\mathrm{theory}}^{E}$, and $d_{\mathrm{theory}}^{C}$. The theoretical and computed densities are also compared in Figure 7. The SPACEBALL calculation results compare much better with the theoretical densities than the densities computed under the spherical and ellipsoidal approximations.

**Figure 8:**
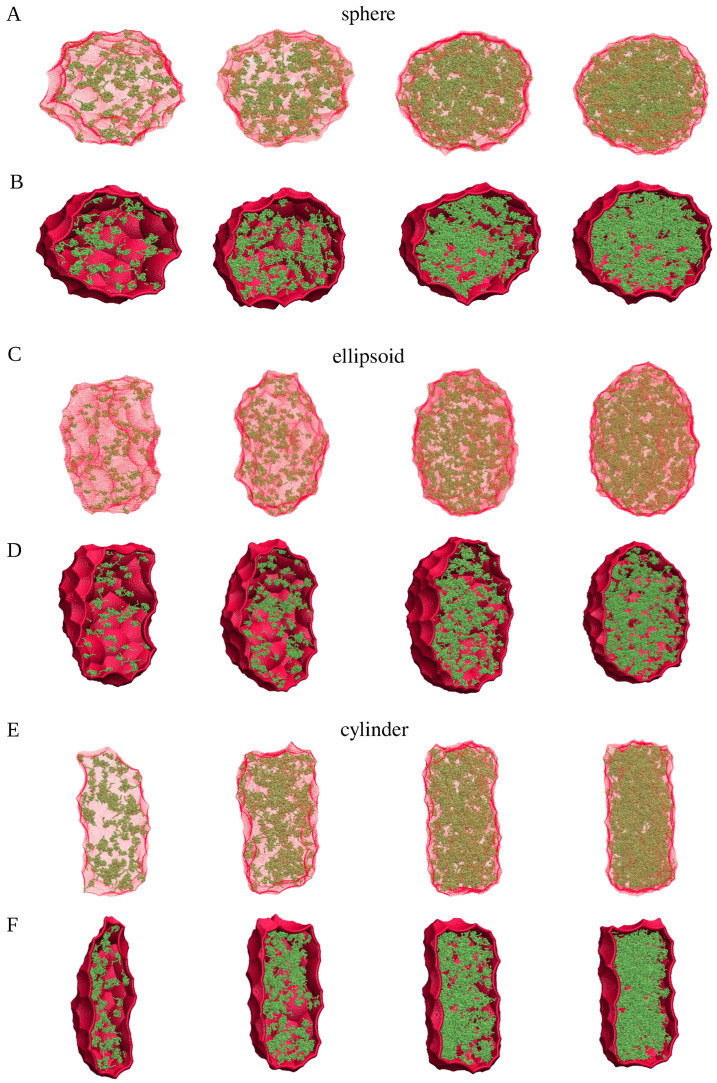
Snapshots of the spherical (**A**,**B**), ellipsoidal (**C**,**D**) and cylindrical (**E**,**F**) clusters with 100 (first column), 200 (second column), 400 (third column), and 600 (fourth column) chains (green), together with envelopes (red) enclosing the clusters. The envelopes are generated by SPACEBALL and shown as semi-transparent surfaces in panels A, C, and E. For comparison, panels B, D, and F show cross-sections of the clusters with opaque envelopes.

Figure 8 shows snapshots of the spherical, ellipsoidal and cylindrical clusters with 100, 200, 400, and 600 chains. The α-synuclein chains are depicted in green, whereas envelopes of these clusters—as determined by SPACEBALL with the optimal probe radii listed in Table 1—are marked in red. For clarity, the envelopes are shown as semi-transparent surfaces in the top panels, whereas the bottom panels show cross-sections through the clusters with opaque envelopes. These snapshots together demonstrate that, firstly, the shape of each of the clusters is clearly and precisely delineated by the SPACEBALL envelopes and, secondly, the envelopes comprise no artificial cavities or pockets. If the selected probe radii were smaller, the wall probes would likely penetrate the cluster interior, creating artificial cavities, and if the selected probe radii were larger, the envelopes would contain artificial pockets, thereby artificially enlarging the computed volumes. Thus, our method for identifying the optimal radius of the wall probes for the SPACEBALL calculation leads to meaningful shapes and volumes for the clusters.

**Figure 9:**
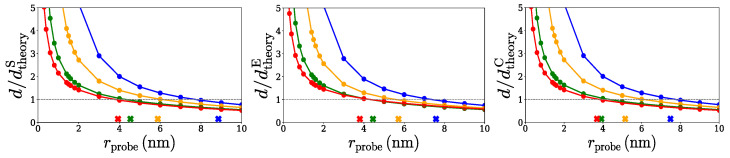
The ratio of the computed density and the theoretical density versus the radius of the wall probes applied in the SPACEBALL computation to the spherical, ellipsoidal, and cylindrical clusters with 100 (data points in blue), 200 (data points in yellow), 400 (data points in green), and 600 (data points in red) chains. The cross symbols (×) indicate the values of the optimal probe radius $r_{\mathrm{probe}}^{O}$ presented in Table 1. The horizontal dashed lines indicate $d=d_{\mathrm{theory}}^{S}$, $d=d_{\mathrm{theory}}^{E}$ and $d=d_{\mathrm{theory}}^{C}$, i.e., perfect agreement between the computed and theoretical densities.

Another way to test our method for selecting the optimal probe radius is to run SPACEBALL in series with different probe radius values and compare the resulting densities *d* of the spherical, ellipsoidal, and cylindrical clusters with the theoretical densities $d_{\mathrm{theory}}^{S}$, $d_{\mathrm{theory}}^{E}$, and $d_{\mathrm{theory}}^{C}$ of these clusters. Figure 9 shows the density rations $d/d_{\mathrm{theory}}^{S}$, $d/d_{\mathrm{theory}}^{E}$, and $d/d_{\mathrm{theory}}^{C}$ versus the probe radius, $r_{\mathrm{probe}}$, for the three sets of clusters. The lines in blue, yellow, green, and red correspond to the clusters comprising 100, 200, 400, and 600 chains. The cross symbols (×) indicate the optimal values of the wall probes, $r_{\mathrm{probe}}^{O}$, presented in Table 1. As can be seen in Figure 9, the values of $r_{\mathrm{probe}}^{O}$ compare well with the corresponding radii of the wall probes at which the computed and theoretical densities are equal, $d=d_{\mathrm{theory}}^{S}$, $d=d_{\mathrm{theory}}^{E}$, and $d=d_{\mathrm{theory}}^{C}$, which further confirms that our choice of optimal probe radii is reasonable.

Another important conclusion that follows from the results shown in Figure 9 is that small variations in the probe radii around the $r_{\mathrm{probe}}^{O}$ values cause only marginal changes in the computed densities of the clusters. This means that even if the optimal probe radii listed in Table 1 are not perfectly accurate, the densities of the clusters are expected to be captured reasonably well using the SPACEBALL method.

**Figure 10:**
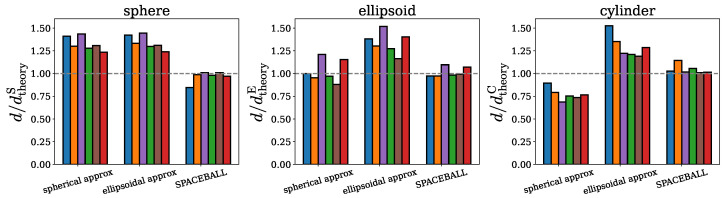
Comparison of the densities obtained using the spherical approximation, the ellipsoidal approximation, and the SPACEBALL method. The ratios of the computed and theoretical densities, $d/d_{\mathrm{theory}}^{S}$, $d/d_{\mathrm{theory}}^{E}$ and $d/d_{\mathrm{theory}}^{C}$, are shown for the spherical, ellipsoidal and cylindrical clusters with 100 (bars in blue), 200 (bars in orange), 300 (bars in purple), 400 (bars in green), 500 (bars in brown) and 600 (bars in red) chains. The dashed lines indicate a perfect agreement between the computed and theoretical densities, i.e., $d=d_{\mathrm{theory}}^{S}$, $d=d_{\mathrm{theory}}^{E}$ and $d=d_{\mathrm{theory}}^{C}$.

Although the SPACEBALL calculations are computationally more demanding and time-consuming than the calculations based on the approximate methods introduced in Section 2.1 and Section 2.2, they yield results superior to those obtained under the spherical and ellipsoidal approximations. The advantage of the SPACEBALL method over the approximate methods is illustrated in Figure 10, where the ratios of the computed and theoretical densities, $d/d_{\mathrm{theory}}^{S}$, $d/d_{\mathrm{theory}}^{E}$, and $d/d_{\mathrm{theory}}^{C}$, are shown for the spherical, ellipsoidal, and cylindrical clusters with 100, 200, 300, 400, 500, and 600 chains. Clearly, in all of the cases studied here, the densities obtained using SPACEBALL with the optimal probe radius $r_{\mathrm{probe}}^{O}$ are closer to the theoretical densities than the densities estimated within the spherical and ellipsoidal approximations.

## 3. Discussion

Having optimized the SPACEBALL-based method with protein clusters of well-defined shapes, as generated using Packmol software (see Section 4.3), we now apply this method to the α-synuclein clusters obtained from the coarse-grained MD simulations (see Section 4.2). The clusters selected for the density calculation are quite diverse and have rather irregular shapes. In the clusters called ‘large cluster A’ and ‘large cluster B’, obtained for a high temperature, the α-synuclein chains are assembled rather loosely. In the clusters called ‘small cluster A’ and ‘small cluster B’, obtained for a low temperature, the chains are packed more densely. And the clusters called ‘medium cluster A’ and ‘medium cluster B’, obtained for a medium temperature, have an intermediate degree of chain packing.

**Figure 11:**
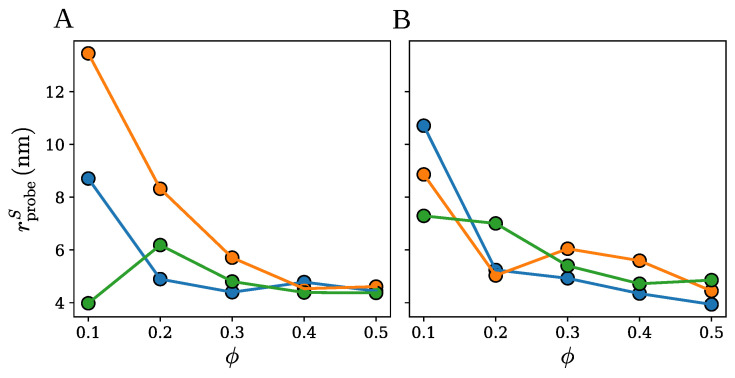
The surface radius $r_{\mathrm{probe}}$ *versus* the surface chain fraction ϕ for the clusters of small (data points in blue), medium (data points in orange), and large (data points in green) size. Panels (**A**,**B**) correspond to the two sets of clusters.

To compute the density of each of the clusters using SPACEBALL, the optimal radius $r_{\mathrm{probe}}^{O}$ of the wall probes needs to be selected. Figure 11 shows how the surface radius $r_{\mathrm{probe}}^{S}$ of the wall probes varies with the surface chain fraction ϕ for the small, medium, and large clusters of group A and B. Importantly, for any of the six clusters, $r_{\mathrm{probe}}^{S}$ is observed not to change significantly as ϕ is varied between 0.3 and 0.5. This observation implies that the SPACEBALL calculation results should be robust and practically unaffected by the exact value of ϕ, as long as it is in the range between 0.3 and 0.5. Therefore, in the following, we use ϕ=0.5 for the selection of the optimal radius of the wall probes for the SPACEBALL calculation. In other words, we take $r_{\mathrm{probe}}^{O}=r_{\mathrm{probe}}^{S}\left(\phi =0.5\right)$.

Table 2 lists the values of $r_{\mathrm{probe}}^{O}$ for the small, medium, and large clusters of group A and B. These optimal probe radii range from about 3.9 to 4.9 nm and, thus, are comparable to or even larger than the radius of gyration of the single chains of α-synuclein forming the clusters. This observation suggests that all of these clusters are rather loose—at least on their surface—and contain space for α-synuclein chains to diffuse and change conformation due to thermal fluctuations.

**Figure 12:**
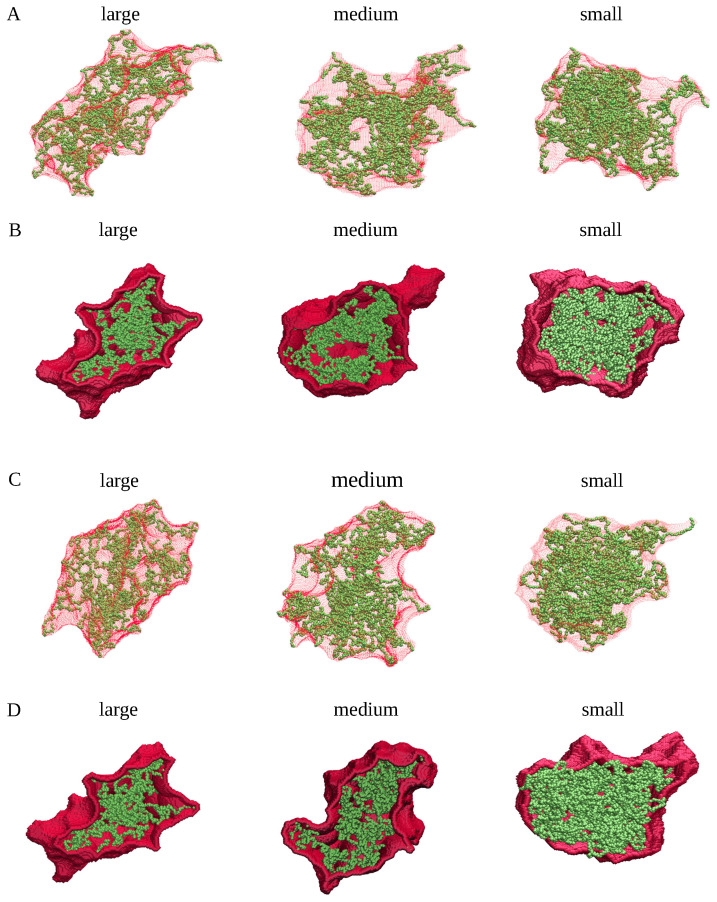
Snapshots of the large (first column), medium (second column), and small (third column) clusters of α-synuclein chains (green), together with envelopes (red) enclosing the clusters. The envelopes are generated by SPACEBALL and shown as semi-transparent surfaces in panels (**A**,**C**). For comparison, panels (**B**,**D**) show cross-sections through the clusters with opaque envelopes.

Table 2 also provides the densities of the small, medium, and large clusters, computed using SPACEBALL with the specified values of $r_{\mathrm{probe}}^{O}$. The corresponding envelopes of these clusters are presented in Figure 12. In panels A and C, the cluster envelopes are shown as semi-transparent surfaces. For comparison, panels B and D show cross-sections through the clusters with opaque envelopes. Each of these envelopes is seen to comprise no artificial overhangs, cavities or pockets. Moreover, the cluster shapes are captured well by the envelopes generated by SPACEBALL with the optimal probe radii.

Interestingly, the computed densities of the small, medium, and large clusters do not differ much, with all falling in a range between 0.22 and 0.31 res/${\mathrm{nm}}^{3}$. The small clusters, generated in the MD simulations at the lowest temperature, were found to have a density of about 0.3 res/${\mathrm{nm}}^{3}$. On the other hand, the large and medium clusters were found to have a lower density of about 0.25 res/${\mathrm{nm}}^{3}$ on average. These results indicate that the densities of the simulation-derived clusters originate from inter- and intra-molecular interactions captured in the coarse-grained MD simulations. These results should thus provide a reliable estimate for the density of the condensed phase of α-synuclein.

**Figure 13:**
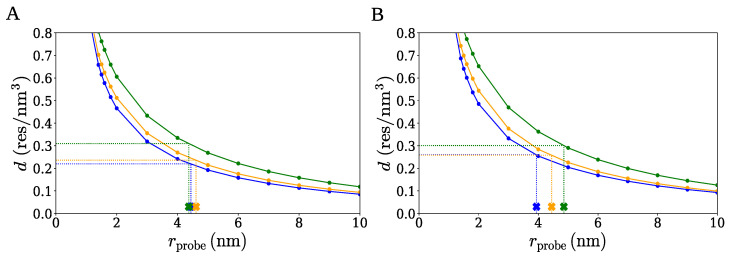
Density of the large (data points in blue), medium (data points in orange), and small (data points in green) clusters of α-synuclein chains *versus* the radius of the wall probes used in the SPACEBALL calculation. Panels (**A**,**B**) correspond to the two sets of clusters. The cross symbols (×) indicate the values of the optimal probe radius $r_{\mathrm{probe}}^{O}$ listed in Table 2.

It should be noted that even if the optimal probe radii listed in Table 2 are not perfectly accurate, the densities of the clusters are expected to be captured reasonably well using the SPACEBALL-based method. This conclusion can be drawn from the data presented in Figure 13, where the density of each of the clusters is shown as a function of the radius $r_{\mathrm{probe}}$ of the wall probes used in the SPACEBALL calculation. The optimal probe radii from Table 2 are marked as cross symbols (×). It can be seen from the curves in Figure 13 that small variations in the probe radii around $r_{\mathrm{probe}}^{O}$ cause only small changes in the cluster density. In other words, if the optimal probe radii were changed by, say, 20%, the computed densities of the clusters would be affected only to a small degree.

**Figure 14:**
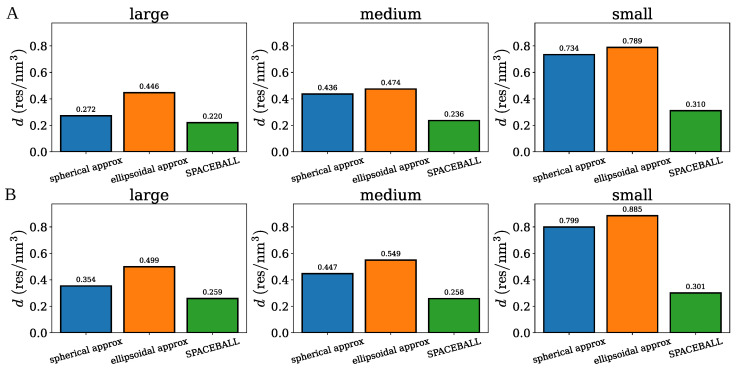
The densities of the large, medium, and small clusters determined using the spherical approximation (bars in blue), the ellipsoidal approximation (bars in orange), and the SPACEBALL method (bars in green). Panels (**A**,**B**) correspond to the two sets of clusters.

The three methods of cluster density calculation are compared in Figure 14, which displays the density of each of the small, medium and large clusters. The bars in blue, orange, and green correspond to the spherical, ellipsoidal, and SPACEBALL-based methods. Notably, the spherical and ellipsoidal approximations yield densities larger than those determined by SPACEBALL, especially for the small clusters with a large degree of chain packing. This result is understandable because the approximated spherical and ellipsoidal volumes do not capture the actual volumes of the simulation-derived clusters with irregular shapes and non-uniform chain distributions, which can be clearly seen in Figure 1 and Figure 3, where a medium-size cluster from the coarse-grained MD simulations is approximated by a uniform sphere and a uniform ellipsoid, respectively. In these examples, both the sphere and the ellipsoid fail to encompass certain regions of the cluster, underestimating the actual volume occupied by the cluster and, thus, overestimating its density.

## 4. Materials and Methods

### 4.1. MD Simulations

To generate protein nanodroplets, or clusters, for testing methods for density calculations, we performed coarse-grained MD simulations of fifty chains of α-synuclein. The initial conformations of these chains were obtained from all-atom implicit-solvent MD simulations of a single molecule of α-synuclein, which were conducted using the generalized Born (GB) model with the NAMD software [43]. The electrostatic screening length corresponding to a monovalent ion concentration of 0.3 M was used to reduce computational costs without significantly compromising solvation effects [44]. An NMR structure of micelle-bound α-synuclein (PDB ID: 1XQ8 [24]) was used as input. The system was subjected to energy minimization to remove steric clashes and stabilize the structure prior to equilibration. This was followed by 1 ns of equilibration at a temperature of 298 K, maintained through a Langevin thermostat with a damping coefficient of $1\;{\mathrm{ps}}^{-1}$. The simulation employed the CHARMM36 force field. Non-bonded interactions were handled using a cutoff of 1.4 nm, a switching distance of 1.3 nm, and a pair list distance of 1.6 nm to ensure accurate treatment of long-range forces. A time step of 2 fs was used. Rigid bonds were applied to hydrogen atoms to maintain numerical stability. Following the equilibration, a production run was carried out for a total simulation time of 3 μs. From this all-atom implicit-solvent MD trajectory, the fifty most extended conformations were selected for subsequent coarse-grained MD simulations.

The coarse-grained MD simulations were conducted using the dynamic structure-based (DSB) model with a non-radial multi-body pseudo-improper-dihedral potential and implicit solvent [45]. In the DSB, amino acid residues are represented by single beads. The dynamics of the beads are derived using the Langevin equation. Temperature is expressed in units of $\epsilon /k_{B}$, where $k_{B}$ is the Boltzmann constant and ϵ represents the energy scale, which is in the order of 1.5 kcal/mol. The time unit τ≈1 ns corresponds to 200 simulation steps.

**Figure 15:**
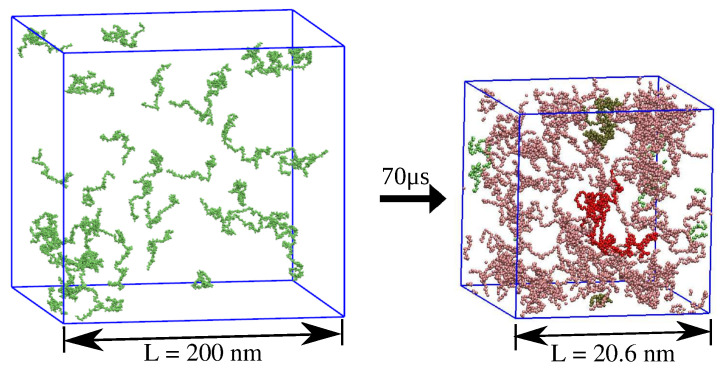
Cluster formation during the coarse-grained MD simulation of fifty chains of α-synuclein. The left panel shows the initial configuration of the simulation system, where the fifty chains of α-synuclein are distributed randomly in the cubic box of side length L=200 nm. The right panel displays the system configuration after 70 μs of MD with the fifty chains of α-synuclein in a cubic box of side length L=20.6 nm. Clusters of α-synuclein, identified by the method described in Section 4.2, are highlighted in different colors.

We used the $P^{-}\;F\;MJ_{0.1}\;C$ variant of the DSB model with the force-field parameters and the damping coefficient, as introduced by Mioduszewski et al. [45]. Fifty chains of α-synuclein, obtained from the all-atom implicit-solvent MD simulations with the GB model, were placed randomly inside a cubic box using the Packmol software [40], as shown in the left panel of Figure 15. The initial density of the system was smaller than 0.001 beads per ${\mathrm{nm}}^{3}$, ensuring that each of the chains did not come into contact with any other. The system was pre-equilibrated for 100 τ, and then the simulation box was compressed uniformly in all three directions at a rate of 0.002 nm/τ until a target box size was achieved. Next, the fifty chains of α-synuclein were simulated at constant volume in a cubic box of side length *L* with periodic boundary conditions. The simulations were carried out with three different box lengths *L* and temperatures *T* over a sub-millisecond timescale, as specified in Table 3. The right panel of Figure 15 shows a snapshot from the simulation with L=20.6 nm taken at the time instance t=70μs.

### 4.2. Cluster Analysis of the Coarse-Grained MD Trajectories

Each of the coarse-grained simulations provided a set of configurations of the system comprising fifty chains of α-synuclein. In order to select clusters of α-synuclein chains for subsequent density calculations, the periodic boundaries used in the MD simulations were unwrapped and agglomerative clustering was applied to each of the configurations obtained from the coarse-grained MD simulations. At the beginning of the clustering procedure, each of the α-synuclein chains in a given configuration was taken as a single cluster. Then the clusters were merged successively. Two clusters were merged if any chain from one cluster was identified to make at least one inter-molecular contact with any chain from the other cluster. The clustering procedure ended when no more clusters could be merged in a given configuration. The agglomerative clustering was applied successively to each of the configurations individually, typically identifying several clusters of different sizes and a number of individual, unassociated chains.

An important factor in the clustering algorithm is the criterion for inter-molecular contacts. Here, we applied a simple distance criterion with the effective radii of amino acid residues used previously in one-bead-per-residue models of intrinsically disordered proteins [46,47]. Namely, we assumed that two beads with indices *i* and *j* and the effective radii $\sigma_{i}$ and $\sigma_{j}$ were in contact if their distance was smaller than a cut-off distance $d_{\mathrm{cut}-\mathrm{off}}=0.75\;\left(\sigma_{i}+\sigma_{j}\right)$ [48].

The clustering of the configurations obtained from the coarse-grained MD simulations allowed us to identify a huge number of α-synuclein clusters of various sizes and shapes. Typically, clusters formed in the MD simulations with low temperatures ($T=0.45\;\epsilon /k_{B}$ or $T=0.5\;\epsilon /k_{B}$) appeared to be somewhat compact, whereas clusters obtained from the MD simulations with the high temperature ($T=1.2\;\epsilon /k_{B}$) were found to be rather loose and irregular in shape.

**Figure 16:**
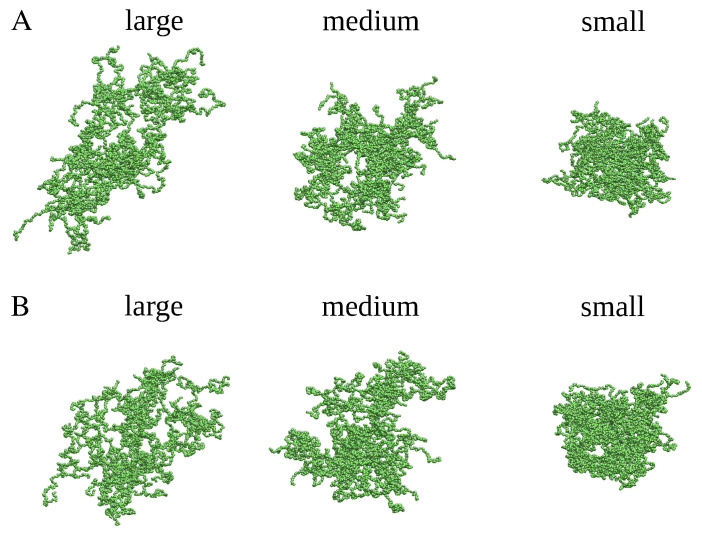
Snapshots of the large, medium, and small clusters selected for assessment of different methods of density calculation. Each of the clusters contains fifty chains of α-synuclein (green). The gyration radius and the maximum diameter of each of the clusters is presented in Table 4. Panels (**A**,**B**) correspond to the two sets of clusters described in the main text and indicated in Table 4. Each of the clusters was generated in the coarse-grained MD simulations and identified by agglomerative clustering with the cut-off distance $d_{\mathrm{cut}-\mathrm{off}}=0.75\;\left(\sigma_{i}+\sigma_{j}\right)$, as described in the main text.

To proceed with testing different methods for density calculation at the supra-molecular level, we randomly selected several distinct clusters with different shapes and volumes, each comprising fifty chains of α-synuclein. The spatial dimensions of the selected clusters were quantified by their radius of gyration, $R_{g}$, and their maximum diameter $D_{\max}$. Specifically, from the MD simulations at a high temperature ($T=1.2\;\epsilon /k_{B}$) we selected two clusters (denoted herein as ‘large cluster A’ and ‘large cluster B’) of comparable spatial dimensions and of very different shapes (see Table 4 and Figure 16). Next, from the MD simulations at the intermediate temperature ($T=0.5\;\epsilon /k_{B}$), we selected two clusters (denoted as ’medium cluster A’ and ’medium cluster B’) with almost identical $R_{g}$ and $D_{\max}$ values and with clearly different shapes (see Table 4 and Figure 16). Finally, from the MD simulations at the low temperature ($T=0.45\;\epsilon /k_{B}$) we selected two clusters (denoted as ’small cluster A’ and ’small cluster B’) that had similar spatial dimensions and rather distinct molecular arrangements (see Table 4 and Figure 16). As quantified by their $R_{g}$ and $D_{\max}$ values, the large clusters were more extended than the medium-size clusters, whereas the small clusters were more compact than the medium-size clusters (see Table 4). With this selection of diverse clusters, our goal is a comprehensive examination of three methods for density calculation of protein clusters.

### 4.3. Generation of Spherical, Ellipsoidal, and Cylindrical Clusters

In principle, the aforementioned goal seems rather straightforward to achieve. However, a major obstacle lies in the absence of reference density values against which the calculated results can be validated. Another difficulty arises from the highly irregular shapes of the α-synuclein clusters obtained from the coarse-grained MD simulations, as shown in Figure 16, for which no unambiguous method of density calculation currently exists; the development of such a method is actually the main objective of this work. Consequently, comparing different density calculation approaches with only the α-synuclein clusters identified in the coarse-grained MD simulations would have a rather limited significance.

**Figure 17:**
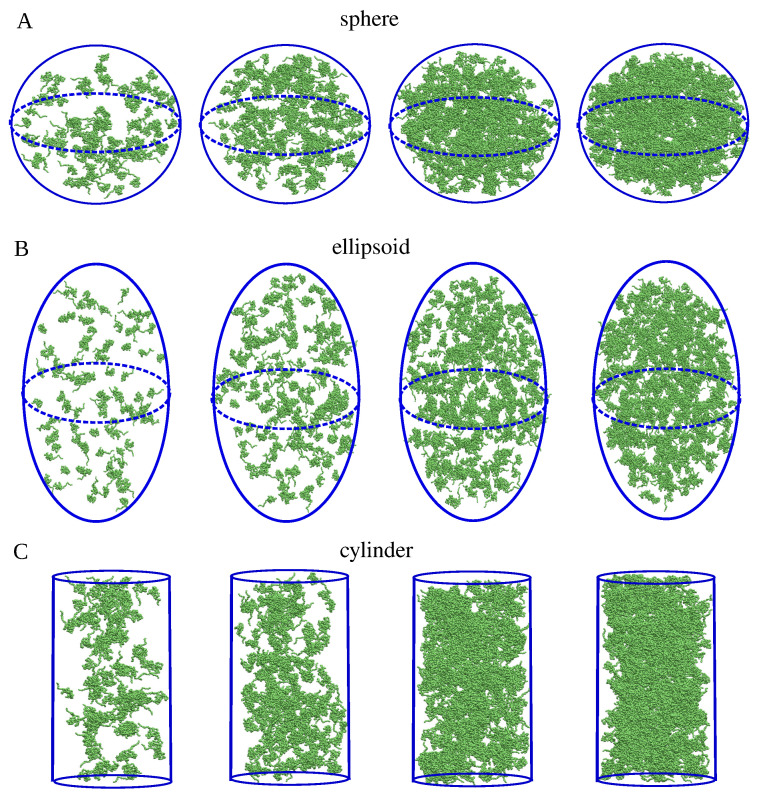
Snapshots of twelve clusters of α-synuclein chains (green) generated using Packmol and used for the assessment of different methods of density calculation. The clusters are shaped like a sphere (**A**), an ellipsoid (**B**), and a cylinder (**C**), and comprise 100, 200, 400, and 600 chains of α-synuclein (from the left to the right).

To address these issues, we generated clusters with well-defined shapes and volumes, as shown in Figure 17. Firstly, we determined the average radius of gyration of the α-synuclein structures obtained from the all-atom MD simulations. Then, we selected an α-synuclein structure whose radius of gyration was closest to the average value. Next, we used the Packmol software [40] to distribute multiple copies of the selected structure within three geometric shapes—a sphere, an ellipsoid, and a cylinder—whose volumes could be calculated using standard geometric formulas. As a result, we obtained systems consisting of 100, 200, 300, 400, 500, and 600 copies of the selected structure with a minimum interatomic distance of 0.2 nm, preventing molecular clashes or overlaps. The generated sphere had a radius of $R_{S}=35$ nm. The ellipsoid had principal semi-axes lengths of $R_{x}=25$ nm, $R_{y}=35$ nm and $R_{z}=50$ nm along the *x*-, *y*-, and *z*-axes, respectively. The cylinder was aligned along the *x*-axis and had a radius of $R_{C}=25$ nm and a height of $H_{C}=93$ nm. The sphere, the ellipsoid, and the cylinder thus had comparable volumes. With *N* denoting the total number of residues forming the cluster, the theoretical density for the spherical clusters was determined using $d_{\mathrm{theory}}^{S}=3N/\left(4\pi R_{S}^{3}\right)$. For the cylindrical clusters, the theoretical density was calculated as $d_{\mathrm{theory}}^{C}=N/\left(\pi R_{C}^{2}H\right)$. For the ellipsoidal clusters, the theoretical density was determined using $d_{\mathrm{theory}}^{E}=3N/\left(4\pi R_{x}R_{y}R_{z}\right)$. We used these systems as a reference for testing density calculation methods, as described in the Results.

## 5. Conclusions

Liquid droplets of IDPs observed in microscopy experiments are spherical and at least several micrometers in diameter [49]. In contrast, protein nonodroplets in MD simulations are at least two orders of magnitude smaller and comprise, at most, a few hundred IDPs [50]. As a result, such IDP nanodroplets have irregular shapes and ambiguous boundaries; thus, their densities are challenging to compute and compare with phase diagrams that are determined experimentally.

We implemented and tested three methods for computing the densities of protein nanodroplets, or clusters, obtained from MD simulations or other molecular modeling techniques. Two of them are based on simple geometrical considerations, as discussed in Section 2.1 and Section 2.2, and involve no adjustable parameters. Our tests indicate that these methods only perform well for homogeneous and globular clusters, whose shapes can be reasonably approximated by spheres or ellipsoids, and fail to correctly capture the volume of clusters with irregular shapes containing protrusions, cavities, or overhangs. In addition, their underlying assumption of uniform mass distribution within clusters leads to density overestimations, as can be seen in Figure 2 and Figure 4. Nevertheless, these methods can be useful in some applications because of their computational efficiency. The third method is based on the SPACEBALL algorithm, which is computationally more demanding and time-consuming than the other methods, but is capable of accurately determining the volumes of clusters of practically any shape, as discussed in Section 2.3. A central parameter of the SPACEBALL algorithm, which governs the resolution of surface and volume detection, is the radius of volume probes. We devised a robust way of optimizing the probe radius for any given protein cluster. The optimization is based on the spatial distribution of protein chains on the cluster surface, and leads to satisfactory accuracy in density calculations using SPACEBALL, as can be seen in Figure 7 and Figure 10. Python scripts for optimizing the probe radius for any given protein nanodroplet, as well as input parameters for the subsequent calculations of the nanodroplet density using SPACEBALL, are deposited on the GitHub platform; see https://github.com/midhunanila/SPACEBALL_DENSITY (accessed on 2 September 2025). It is worth noting at this point that our procedure for the probe radius optimization may fail or lead to unsatisfactory results in cases of very small clusters, when distinguishing between protein chains on the cluster surface and inside the cluster is impossible. Nevertheless, the SPACEBALL-based method yields generally superior results to those obtained under the spherical and ellipsoidal approximations.

The α-synuclein clusters obtained from the coarse-grained MD simulations with different temperatures differ in both their morphology and packing degree, as illustrated in Figure 16 and Figure 12. The SPACEBALL-based analysis presented in Section 3 revealed, however, that the densities of these clusters fall in a rather narrow range between 0.22 and 0.31 res/${\mathrm{nm}}^{3}$. The small clusters, generated in the MD simulations at lower temperatures, are found to have a density of about 0.3 res/${\mathrm{nm}}^{3}$. The larger clusters, generated in the MD simulations at higher temperatures, are found to have a lower density of about 0.25 res/${\mathrm{nm}}^{3}$ on average. Importantly, it should be noted that these densities are much lower than the average density of a folded protein, which is estimated to be about 7 res/${\mathrm{nm}}^{3}$ [51]. Therefore, even the α-synuclein clusters produced at low temperatures are rather loose and comprise substantial amounts of solvent.

Here, we used the coarse-grained DSB model to generate clusters of α-synuclein chains. It should be noted, however, that the methods of computing nanodroplet density discussed in Section 2.1, Section 2.2 and Section 2.3 are independent of the simulation models or force fields used to generate the nanodroplet structures. Our density calculation methods are applicable to protein nanodroplets obtained from all-atom or coarse-grained MD simulations with any force field or using any other molecular modeling technique.

Recently, the LLPS of α-synuclein has been studied in MD simulations employing the Martini force field [41]. Fifty chains of α-synuclein were simulated at different conditions in a cubic box with a side length ranging from about 48 to 66 nm. The concentration of α-synuclein within nanodroplets was determined using a variant of the ellipsoidal approximation. The average concentration of α-synuclein in the simulated nanodroplets was found to be about 30 mM, which is an order of magnitude higher than the ∼0.25 res/${\mathrm{nm}}^{3}$ (corresponding to the α-synuclein concentration of about 3 mM) that we report in Table 2. This comparison indicates that our estimation of α-synuclein concentration in nanodroplets, i.e., about 3 mM, may be closer to the experimentally determined values [34] than the concentration of about 30 mM obtained from the Martini simulations [41].

## Figures and Tables

**Table 1:** The optimal probe radii $r_{\mathrm{probe}}^{O}$ alongside the SPACEBALL-computed densities of the spherical, ellipsoidal, and cylindrical clusters with 100, 200, 300, 400, 500, and 600 chains.

*n*	$r_{\mathrm{probe}}^{O}$ (nm)	*d* (res/${\mathrm{nm}}^{3}$)	$d_{\mathrm{theory}}^{S}$ (res/${\mathrm{nm}}^{3}$)
100	8.86	0.066	0.078
200	5.88	0.154	0.156
300	5.07	0.236	0.234
400	4.54	0.306	0.312
500	4.06	0.394	0.390
600	3.93	0.454	0.468
*n*	$r_{\mathrm{probe}}^{O}$ (nm)	*d* (res/${\mathrm{nm}}^{3}$)	$d_{\mathrm{theory}}^{E}$ (res/${\mathrm{nm}}^{3}$)
100	7.58	0.074	0.076
200	5.72	0.148	0.152
300	4.74	0.250	0.228
400	4.45	0.299	0.304
500	3.82	0.377	0.380
600	3.80	0.488	0.456
*n*	$r_{\mathrm{probe}}^{O}$ (nm)	*d* (res/${\mathrm{nm}}^{3}$)	$d_{\mathrm{theory}}^{C}$ (res/${\mathrm{nm}}^{3}$)
100	7.49	0.078	0.076
200	5.15	0.175	0.153
300	4.68	0.234	0.230
400	3.92	0.323	0.306
500	3.95	0.387	0.383
600	3.69	0.467	0.460

**Table 2:** The optimal probe radius $r_{\mathrm{probe}}^{O}$ and the computed density of the clusters obtained from the coarse-grained MD simulations.

Cluster Set	Cluster Extension	$r_{\mathrm{probe}}^{O}$ (nm)	*d* (res/${\mathrm{nm}}^{3}$)
A	large	4.37	0.220
	medium	4.61	0.236
	small	4.44	0.310
B	large	4.86	0.259
	medium	4.45	0.258
	small	3.94	0.301

**Table 3:** Parameters of the coarse-grained MD simulations of fifty chains of α-synuclein.

Temperature *T* ($\epsilon /k_{B}$)	Box Length *L* (nm)	Simulation Time (μs)
0.45	18.5	481
0.50	20.6	487
1.20	26.1	313

**Table 4:** Dimensions of the α-synuclein clusters derived from the coarse-grained MD simulations and selected for an assessment of density calculation methods. Each of the clusters is formed of fifty chains. The cluster size (large, medium, or small) is indicated in the second column and quantified by the gyration radius ($R_{g}$) and the maximum diameter ($D_{\max}$) in the third and fourth column, respectively. The simulation temperature (*T*) is given in the fifth column.

Cluster Set	Cluster Extension	$R_{g}$ (nm)	$D_{\max}$ (nm)	*T* ($\epsilon /k_{B}$)
A	large	14.2	58.6	1.2
	medium	12.1	44.3	0.5
	small	10.2	36.7	0.45
B	large	13	52.3	1.2
	medium	12	44.3	0.5
	small	9.9	38.7	0.45

## Data Availability

The original data presented in the study are openly available in Repository for Open Data (RepOD) at https://doi.org/10.18150/GE0ZEB.
